# Biology and Treatment Advances in Cutaneous Squamous Cell Carcinoma

**DOI:** 10.3390/cancers13225645

**Published:** 2021-11-11

**Authors:** Alesha A. Thai, Annette M. Lim, Benjamin J. Solomon, Danny Rischin

**Affiliations:** 1 Department of Medical Oncology, Peter MacCallum Cancer Centre, 305 Grattan St., Parkville, Melbourne, VIC 3000, Australia; annette.lim@petermac.org (A.M.L.); ben.solomon@petermac.org (B.J.S.); danny.rischin@petermac.org (D.R.); 2 The Sir Peter MacCallum Department of Oncology, University of Melbourne, Melbourne, VIC 3000, Australia

**Keywords:** cutaneous squamous cell carcinoma, CSCC, treatment, advances, biology, immunocompromised, immune checkpoint inhibition, immunotherapy

## Abstract

**Simple Summary:**

Skin cancers are the most diagnosed type of cancer worldwide. Cutaneous squamous cell carcinoma—a type of skin cancer—usually affects older people who have chronic sun exposure, as well as people with weakened immune systems. There has been significant recent progress in the treatment of this type of cancer with immune checkpoint inhibitors that utilize the immune system to target cancer. In concert with advances in treatment, our understanding of the biology of skin cancer has also deepened. The authors have reviewed the risk factors, biology, and advances in treatment in this publication.

**Abstract:**

Cutaneous squamous cell carcinoma (CSCC) is the second most common skin cancer diagnosed worldwide. CSCC is generally localized and managed with local therapies such as excision and/or radiotherapy. For patients with unresectable or metastatic disease, recent improvements in our understanding of the underlying biology have led to significant advancements in treatment approaches—including the use of immune checkpoint inhibition (ICI)—which have resulted in substantial gains in response and survival compared to traditional cytotoxic approaches. However, there is a lack of understanding of the biology underpinning CSCC in immunocompromised patients, in whom the risk of developing CSCC is hundreds of times higher compared to immunocompetent patients. Furthermore, current ICI approaches are associated with significant risk of graft rejection in organ transplant recipients who make up a significant proportion of immunocompromised patients. Ongoing scientific and clinical research efforts are needed in order to maintain momentum to increase our understanding and refine our therapeutic approaches for patients with CSCC.

## 1. Introduction

CSCC is the second most common skin cancer diagnosed worldwide [1]. Important risk factors for CSCC include ultraviolet (UV) radiation and immunosuppression. Most patients have curable, localized disease, but a small proportion (2–5%) develop unresectable locally advanced or metastatic disease [2,3]. Historically, systemic therapy options for these patients were limited; however, there have been advances in our understanding of the biology of CSCC—particularly, an appreciation of the high tumor mutational burden (TMB) observed in most cases of CSCC, and the role of the immune system in tumor prevention and control. This led to a pivotal study of an immune checkpoint inhibitor (ICI) in patients with metastatic or locally advanced, unresectable CSCC, and changed the treatment paradigm for these patients. Efforts are now underway to assess the benefit of ICI in patients with high-risk localized disease, where the chance for cure with improved neoadjuvant or adjuvant approaches is greater. This review discusses the epidemiology, risk factors, and genomic alterations underlying CSCC, and summarizes treatment advances for CSCC.

## 2. Epidemiology

Non-melanoma skin cancers (NMSCs) comprise of basal-cell carcinomas (BCCs; ~80%), CSCC (~20%), and rarer skin cancers. The incidence of CSCC is likely underestimated, as accurate figures are difficult to ascertain, with significant variation in cancer registry practices between countries as a result of the high incidence, relatively low mortality, and multiplicity of CSCC [4,5]. However, it is clear that NMSCs are the most common types of cancer diagnosed in many regions—including Australia, North America, and Europe—and their associated public health burden is significantly underestimated [4,5,6,7,8]. The incidence of CSCC in patients aged 75 years or older is 5–10 times higher than in their younger counterparts. Men are at higher risk of CSCC than women, which was traditionally hypothesized to be a reflection of higher occupational exposure, but there is some suggestion that differences in sexual biology may be a factor in the observed disparity [9,10,11,12,13]. As UV exposure is the strongest risk factor for CSCC, most cases arise in the head and neck region, where UV exposure is highest.

## 3. Risk Factors

**Figure 1:**
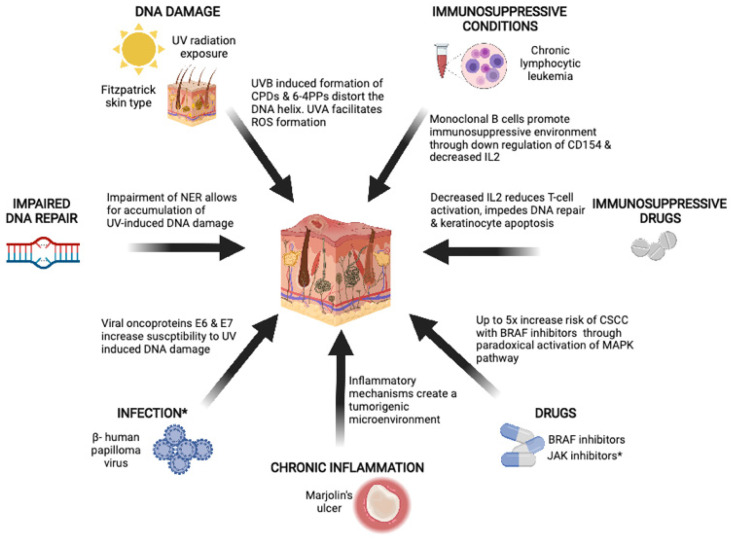
Risk factors for CSCC include UV radiation exposure, immunosuppressive conditions and drugs, inflammations such as those from chronic wounds, and impaired DNA repair. * Infection with β-human papillomavirus and JAK inhibitors are also thought to increase the risk of CSCC, but clear evidence and the underlying biology are not as well established. Created using biorender.com (accessed on 1 September 2021).

The pathogenesis of CSCC is multifactorial. Chronic UV exposure plays an important role, but other risk factors include immunosuppression, environmental exposures, chronic inflammation, and drugs (Figure 1).

### 3.1. Ultraviolet Radiation

Chronic UV exposure is the most important risk factor for CSCC [14]. Sunlight produces three main types of UV radiation: UVA, UVB, and UVC. UVA radiation exposure increases the risk of CSCC, but is less mutagenic than UVB. UVA radiation causes indirect DNA damage by facilitating the formation of reactive oxygen species, which can interact with DNA, lipids, and proteins to form pre-mutagenic adducts [15]. UVB radiation directly damages DNA and RNA by causing the formation of cyclobutane-pyrimidine dimers (CPDs) and 6-4 photoproducts (6-4PPs), which distort the DNA helix, impeding transcription and replication [15]. Particular genomic positions, as a result of their structure, are more vulnerable to UVB-induced DNA damage—for instance, the *TP53* gene, which is the most frequently mutated gene in CSCC [16]. In vivo studies show that mice exposed to chronic UV radiation develop inactivating *TP53* mutations as early as 1 week post-exposure [17]. UVC has the shortest wavelength, and is completely absorbed by the Earth’s ozone layer.

There is marked global variation in CSCC incidence, reflecting not only varying levels of UV exposure, but also genetic propensity to UV damage. The amount of melanin pigment in the skin can be categorized using the Fitzpatrick skin type scale, and is correlated with UV susceptibility and skin cancer risk [18]. Pale or white skin that burns easily and does not tan, classified as Fitzpatrick type 1, has a higher risk of developing skin cancers compared to people with Fitzpatrick type 6 skin, who have very pigmented skin that rarely or never burns [18]. As such, Australia, as a consequence of its location, relative lack of ozone, and high proportion of Anglo-Saxon population, has one of the highest incidences of NMSCs, including CSCC [4,19].

### 3.2. Immunosuppression

The role of the immune system in the development of CSCC has long been recognized from the significantly increased risk of CSCC observed in immunosuppressed patients [20]. Furthermore, the success of ICI (discussed in detail below) highlights the anticancer potency of an intact immune system.

Immunosuppression can be a result of host factors—such as chronic lymphocytic leukemia (CLL) or HIV—or extrinsic factors, such as drugs. This review will address the most common causes of immunosuppression, such as CLL and immunosuppressive drugs.

#### 3.2.1. Chronic Lymphocytic Leukemia

CLL is a low-grade lymphoproliferative malignancy characterized by clonal proliferation of functionally incompetent B cells; it is the most common leukemia in developed countries, accounting for up to 35% of all leukemias [21]. CLL was the most common cause of immunosuppression (34%, *n* = 20/59) in a multicenter retrospective study of patients with CSCC receiving immune checkpoint inhibition [22]. Patients with CLL are 5–8 times more likely to develop CSCC compared to patients without CLL [23,24,25]. Furthermore, the risk of recurrence and CSCC-specific mortality is increased in patients with CLL [26,27]. The risk of metastasis at 5 years has been reported to be 18%, with a standardized mortality ratio of 17.0 (95% CI 14.4–19.8) [27].

CLL is typically diagnosed in older people, with a median age of 70 years. Older age, a higher incidence in men, and the associated immunosuppressive effects of CLL all contribute to the higher risk of CSCC. The strongest risk factor for developing CSCC in the setting of CLL is prior history of any skin cancer, but other CLL risk factors associated with a higher risk of developing CSCC include CLL international prognostic index, Rai stage, and lymphocyte doubling time [28].

The biology underlying the increased risk of CSCC is not fully understood. B cells are traditionally known for antigen presentation, antibody production, and the release of effector cytokines that modulate T-cell responses. There is growing evidence, however, that a newly identified, heterogeneous group of B cells—called regulatory B cells—can modulate the immune response to tumors [29]. In the setting of CLL, monoclonal B cells have been shown to promote an immunosuppressive environment via the downregulation of CD154 in activated T cells in preclinical models. CD154 plays a critical role in stimulating B cells, monocytes, and dendritic cells to differentiate and proliferate [30]. Additionally, in clinical tumor samples, higher levels of interleukin-2 (IL-2) receptors have been detected in patients with CLL compared to patients without CLL. IL-2 receptors are thought to be secreted from T-regulatory cells, and bind free IL-2, thus decreasing its availability [31]. Suppression of IL-2 has been shown to induce CD8+ T-cell anergy [32]. Other immune deficits have been identified in patients with CLL, such as impaired phagocytosis and functional defects in helper B cells [33]. It has also been hypothesized that in addition to the immunosuppressive effects of CLL, shared genetic risk factors between CLL and NMSC can contribute to the association between the two diseases [28,34].

#### 3.2.2. Drugs

A number of drugs are associated with an increased risk of CSCC via different mechanisms, ranging from immunosuppression, to the paradoxical activation of pathways that lead to keratinocyte proliferation and loss of apoptosis.

##### Immunosuppressive Drugs

Long-term immunosuppressive drug regimens are most commonly utilized in organ transplant recipients (OTRs), and involve multiple classes of drugs to minimize graft rejection. As a result, their immunosuppressive effects can be profound, and increase the risk of CSCC by hundreds-fold. In one long-term observational study, approximately 30% of OTRs developed NMSCs, the majority of which were CSCC. The mean time from transplant to first CSCC was 9.9 years, and overall cumulative incidence increased over time to 10.6%, 24.8%, 53.9%, and 73.9% at 5, 10, 20, and 30 years post-transplant, respectively [20]. Patients who have undergone heart or lung transplantations are more susceptible to CSCC formation than renal transplant recipients, likely reflecting the more potent immunosuppressive regimens required for those organs [35,36]. CSCCs developing in OTRs have a higher risk of recurrence, metastases, and cancer-specific death compared to non-transplant patients [37].

Calcineurin inhibitors such as cyclosporine and tacrolimus are a commonly used class of immunosuppressive drugs. They reduce IL-2 production and IL-2 receptor expression, leading to reduced T-cell activation. Cyclosporine, however, also impedes the UV-induced DNA repair mechanisms and keratinocyte apoptosis, by counteracting p53 through ATF3 [38,39]. Furthermore, there is in vitro evidence that cyclosporine can induce epithelial–mesenchymal transition via the upregulation of TGF-β, thus altering the phenotype to a more invasive and aggressive tumor type [40].

Tacrolimus is a more modern calcineurin inhibitor, which has been increasingly used since the 1990s [41]. Interestingly, studies have shown that tacrolimus is not associated with an increased risk of CSCC [42,43], and does not confer resistance to UV-induced apoptosis in keratinocytes—unlike cyclosporine [44].

Since the early 2000s, there has been increasing use of mechanistic target of rapamycin (mTOR) inhibitors—such as rapamycin and sirolimus—as immunosuppressants. mTOR is a protein kinase that plays a role in cell proliferation and survival, as well as modulation of the innate and adaptive immune system [45,46]. Interestingly, however, in vivo studies of mice show that sirolimus—an mTOR inhibitor—significantly delays CSCC development and reduces its multiplicity, even if co-administered with cyclosporine, through inhibition of the transcription factor ATF3 [47,48,49]. ATF3 downregulates expression of *TP53*, which is one of the most commonly mutated genes in CSCC [38,50]. Furthermore, randomized controlled trials (RCTs) also show a significantly reduced incidence of CSCC in patients receiving mTOR inhibitors compared to cyclosporine [51], as well as in patients who were prescribed sirolimus after three months of treatment with cyclosporine (1.2% vs. 4.3%, *p* < 0.001) [43,52].

Oral glucocorticoids are a frequently used immunosuppressant, but data regarding the risk associated with the development of CSCC are inconsistent. Two studies—a case–control study, and a planned sub-study of an RCT—found no association between oral steroid use and risk of CSCC [53,54]. In contrast, a cohort study found that patients on prolonged courses of oral glucocorticoids were at higher risk of developing CSCC (standardized incidence ratio 2.45; 95% CI 1.37–4.04) [55]. Another study of OTRs found that higher cumulative immunosuppression from a combination of cyclosporine, azathioprine, and oral prednisolone increased the risk of CSCC by fourfold compared to lower cumulative doses [56]. However, there was no association between the cumulative doses of each drug alone and risk of CSCC. This highlights the possibility that the overall level and duration of immunosuppression, regardless of agent, is a factor impacting the risk of developing CSCC. Ultimately, it will be difficult to ascertain the true risk of CSCC arising in patients taking a commonly used drug such as oral glucocorticoids. There are many indications for oral glucocorticoids, thus increasing potential confounders and biases. Furthermore, accurate information regarding duration of therapy—which can vary widely, from a few days, to many months or years—is difficult to gather at a population-based level.

##### BRAF Inhibitors

A number of targeted therapies are associated with cutaneous side effects. Squamoproliferative lesions such as actinic keratoses and CSCC are most commonly seen with BRAF inhibitors such as vemurafenib, dabrafenib, and encorafenib, which can be used as monotherapies for patients with metastatic melanoma harboring *BRAF* V600E mutations. A meta-analysis of seven randomized trials found that 18% (95% CI 0.12–0.26) of patients on vemurafenib develop CSCC [57]. In patients taking dabrafenib, CSCC develops in 6–26% of patients [58,59]. BRAF-inhibitor-associated abnormal squamous proliferation is thought to be induced by the paradoxical activation of the mitogen-activated protein kinase (MAPK) pathway, and subsequent ERK-mediated transcription in wild-type BRAF keratinocytes—particularly in the presence of oncogenic RAS mutations [60,61,62,63]. CSCC arising from BRAF inhibition typically occurs within the first 3 months of treatment, and age has been identified as an independent risk factor [64]. Following the establishment of efficacy of BRAF inhibition in metastatic melanoma, overall survival and response with the combination of BRAF with MEK inhibition was found to be superior compared to BRAF monotherapy. Dual blockade of BRAF and MEK is now the standard of care for patients with metastatic *BRAF* V600E mutations. Fortunately, the risk of squamoproliferative lesions—including CSCC—significantly decreased with the addition of MEK inhibition, with a reported incidence of 0–2% [65,66,67].

##### JAK1/2 Inhibitors

Janus kinase (JAK)1/2 inhibitors such as ruxolitinib are used to treat myelofibrosis or polycythemia vera. A number of cases have been reported wherein the initiation of JAK1/2 inhibitors is associated with the development of multiple, rapidly progressing CSCCs [68,69,70]. The incidence of newly diagnosed non-melanoma skin cancer was 17.1% in patients receiving ruxolitinib compared to 2.1% in those receiving best available therapy for myelofibrosis in the long-term follow-up of a phase III RCT [71]. The exact mechanism of tumorigenesis is unknown, but JAK1/2 aberrant hyperactivation has been associated with tumor proliferation and survival in different cancer types [72]. Interestingly, ruxolitinib was shown to reduce tumor progression in in vitro experiments of cyclosporine-induced CSCC cell lines [73].

### 3.3. Marjolin’s Ulcers

Marjolin’s ulcers describe a rare form of CSCC that arises from areas of chronic inflammation such as burn scars, venous stasis ulcers, and pressure sores [74]. Marjolin’s ulcers are more aggressive than spontaneous CSCC, with the risk of recurrence or metastases reported to be approximately 30% in case series [75,76]. There is a long latency period from initial injury to the development of CSCC, with an average time of 30 years reported [74,77,78]. The relationship between inflammation and tumorigenesis has long been appreciated, with examples of cancers arising from patients’ inflammatory bowel disease and *Helicobacter*-induced gastritis [79]. Inflammatory mechanisms ensure appropriate responses to infections, and promote wound healing, but can also create a microenvironment that promotes tumorigenesis via the recruitment of immune cells and subsequent release of cytokines and growth factors [80].

### 3.4. Environmental Exposure

Other environmental risk factors include chronic arsenic exposure, which most commonly occurs from contaminated drinking water [81]. Arsenic-induced CSCC can develop even in non-sun-exposed sites. Ionizing radiation via environmental, therapeutic, or diagnostic exposure is also a known risk factor, although the risk of BCC is higher than that of CSCC, as the basal layer of the epidermis is more affected than more superficial layers [82,83,84]. Occupational exposure to aromatic hydrocarbons such as benzene and mineral oil have also been identified as risk factors for the development of CSCC, and are of particular importance in occupations such as firefighting and petroleum work [85,86].

### 3.5. Inherited Bone Marrow Failure Sydromes (IBMFSs)

IBMFSs comprise of rare diseases typically characterized by genetic mutations resulting in bone marrow failure. These syndromes include Fanconi anemia and dyskeratosis congenita as the most common disorders, which are associated with defects in DNA repair and telomere function, respectively. Patients with these conditions are at increased risk of hematological and solid malignancies due to multiple factors that arise from the genetic disruption, resulting in genomic instability and bone marrow failure. The risk of CSCC is more notable in patients with Fanconi anemia and dyskeratosis congenita. Skin cancers make up approximately 10–20% of cancer cases in patients with Fanconi anemia and dyskeratosis congenita, and typically occur at a median age of approximately 30 years [87,88,89].

### 3.6. Beta Human Papillomavirus

HPV comprises several heterogeneous subgroups; α-papillomavirus (α-HPV) subtypes are associated with mucosal SCCs, such as cervical and oropharyngeal cancer, but it is the β-papillomavirus (β-HPV) subtypes that are hypothesized to be a risk factor for CSCCs. β-HPV was first discovered in the context of patients with a rare skin disorder—epidermodysplasia verruciformis. Patients develop pre-cancerous wart-like lesions that progress to CSCC in UV-exposed areas. Multiple β-HPV types were found in these lesions, thus raising the possibility of the carcinogenic role of β-HPV. Complicating matters, however, is the relative ubiquitousness of β-HPV in the skin. β-HPV DNA is detected in the skin of 39–91% of immunocompetent patients—particularly in hair follicles, which are considered to be a natural reservoir [90,91]. There are, however, multiple factors that suggest that β-HPV may be a risk factor for the development of CSCC.

Firstly, immunocompromised patients have an increased risk of CSCC, and have significantly higher rates of β-HPV infection and higher viral loads, suggesting a potential causal relationship between β-HPV and CSCC [92]. Second, observational studies have shown an association between β-HPV DNA and/or serum antibodies and CSCC in both immunocompromised and immunocompetent patients [93]. A meta-analysis of over 3000 immunocompetent patients found an overall association of β-HPV and CSCC (OR 1.42; 95% CI 1.18–1.72) [94]. Notably, some of these studies incorporated BCC cases, and no associations were observed between β-HPV and BCC [95,96,97]. Thirdly, there are increasing preclinical data supporting the role of β-HPV in tumor initiation, but not necessarily in tumor maintenance [98]. β-HPV DNA is detected at high levels in pre-cancerous lesions such as actinic keratoses, whereas lower levels are detected in CSCC lesions [99,100]. In vitro and in vivo studies have shown that the HPV oncoproteins E6 and/or E7 from HPV types 5, 8, and 38 can increase susceptibility to UV-induced oncogenesis via alterations in p53 and Notch1 signaling [101,102,103,104,105,106,107,108]. β-HPV is also thought to infect and expand adult tissue stem cells, thus enabling cells to persist and accumulate mutations [109]. It is hypothesized that once cells have accumulated mutations such as *TP53* and *Notch*, which allow for ongoing cell proliferation, expression of viral oncogenes becomes redundant, and they are no longer positively selected.

## 4. Biology and Pathogenesis

There have been significant advances in our understanding of the biological pathways in CSCC development, with multiple genes identified as playing a critical role in tumor initiation and persistence. CSCC, however, has one of the highest median TMBs of any tumor type; thus, hundreds of mutations can be found per megabase [110]. One of the challenges in understanding the biological pathways involved in CSCC is separating true oncogenic mutations from passenger mutations. Here, we discuss the oncogenic roles of selected commonly mutated genes such as *TP53*, *Notch*, and *CDKN2A.* No specific oncogenic drivers of CSCC have been identified.

p53 functions predominantly as a transcription factor, and can activate or repress a large number of target genes. In particular, p53 plays an important role in modulating nucleotide excision repair (NER) and other DNA repair pathways that are essential in the repair of UV-induced DNA damage [111]. Mutations in *TP53* allow for ongoing, unrepaired UV-induced DNA damage. As an example, the risk of CSCCs is significantly higher if there are genetically impaired DNA repair mechanisms, such as in patients with xeroderma pigmentosum who develop NMSCs during childhood [112].

Mutations in *TP53* occur early in CSCC development, and are often found in normal keratinocytes [113,114,115] and pre-malignant lesions [116]. Whole-exome sequencing of CSCC has identified bi-allelic *TP53* mutations in nearly all tumors, again suggesting that the loss of wild-type *TP53* is an early step in carcinogenesis [117,118,119]. This is in contrast to other solid malignancies, where *TP53* gene mutations occur later in tumor evolution [120,121,122]. Further evidence of the oncogenic role of *TP53* in CSCC has been shown in in vivo studies, where homozygous p53-knockout mice rapidly developed CSCC after UV exposure [123,124]. p53-mutant cells are more resistant to UV-induced apoptosis, and have a proliferative advantage over wild-type keratinocytes [125].

The Notch signaling pathway is commonly affected, and *Notch* mutations are found in 60–80% of CSCCs [118,119,126]. Notch is a highly conserved intercellular signaling mechanism that plays a critical role in the development and maintenance of tissue homeostasis [127]. Genes of the *Notch* family encode four transmembrane receptors (Notch1–4). In the epidermis, Notch signaling is involved in the terminal differentiation of keratinocytes [128]. Interestingly, *Notch* can have oncogenic or tumor-suppressive functions depending on the cell context [129]. Constitutive Notch1 signaling as a result of activating mutations is the initiating step in almost all T-cell acute lymphoblastic leukemia (T-ALL) cases [130,131]. In contrast, loss of Notch signaling—particularly Notch1 and Notch2—is associated with carcinogenesis in keratinocytes [118,132,133]. Preclinical studies have shown that Notch1 is a downstream positive target of p53 in keratinocytes; thus, inactivating *TP53* mutations can further lead to reduced Notch1 expression [134]. Several in vivo experiments have shown Notch1 deficiency or Notch1 inhibition in mice can result in the spontaneous development of CSCC [135]. A possible mechanism is via upregulation of the Wnt/β-catenin pathway [132]. Intriguingly, Notch deficiency or loss does not purely exert its effect autonomously on cells, but can also create a pro-tumorigenic microenvironment. Loss of Notch signaling disrupts skin barrier function, creating a chronic wound-like environment [136]. As a result, mesenchymal components are recruited for repair, which also stimulates a vascularized and growth-factor-rich stroma, providing an ideal environment for tumor formation [136]. There is also clinical evidence of Notch inactivation resulting in increased CSCC risk. Semagacestat, a γ-secretase inhibitor, was developed as a drug for Alzheimer’s disease. A phase III RCT of semagacestat was halted early due to lack of efficacy as well as an increased risk of CSCC. γ-Secretase, in addition to converting amyloid precursor protein to amyloid-β is also responsible for cleaving and activating Notch1; thus, its inhibition indirectly inactivates Notch1 [137].

The cyclin-dependent kinase inhibitor 2A *(CDKN2A)* gene encodes two tumor-suppressor genes: *p16^INK4a^* and *p14^ARF^.* Both genes regulate cell cycling: *p16^INK4A^* binds to CDK4 and CDK6, thus preventing Rb protein phosphorylation and G1-S phase progression, while *p14^ARF^* binds to MDM2, preventing p53 degradation and Rb inactivation, causing cell arrest. Methylation of the promoter region is the most common mechanism of p16 and p14 inactivation in CSCC, followed by point mutations and loss of heterozygosity [138]. Alterations in *CDKN2A* are found in up to 80% of CSCCs [119]. Inactivating mutations of *CDKN2A* result in uncontrolled cell cycling and proliferation. A recent analysis, however, consistently found upregulation of *CDKN2A* in gene expression profiles and cell lines, in contrast to the pre-existing literature; the authors hypothesized that ERK signaling in CSCC may upregulate *CDKN2A* as a stress response to induce senescence rather than stimulating cell cycling [139].

*RAS* gene mutations are among the most common activating mutations found in human cancers, and also present a significant therapeutic challenge due to their molecular characteristics. The *RAS* gene encodes four RAS proteins: HRAS, NRAS, and two splice variants of KRAS. RAS proteins belong to a family of small GTPases that cycle between “off” and “on” states [140]. Activating *RAS* mutations can result in oncogenic constitutive activation of the RAF-MEK-ERK and PI3K-AKT pathways, leading to cell proliferation [141]. In CSCC, *HRAS* mutations are most common, and are found in 3–20% of CSCCs [118,133]. In keratinocytes, upregulated expression of RAS alone is not sufficient to induce tumorigenesis [142]. Concomitant *Notch1* deletion, IκBα co-expression, or CDK4-mediated bypass of Rb cell cycle restraints increase CSCC formation in the presence of activated RAS [132,143,144].

## 5. Tumor Mutation Burden

As a result of the chronic nature of UV exposure and the mechanism of DNA damage, there are cumulative DNA aberrations in CSCCs. In a study examining TMB in over 100,000 tumor samples, CSCC had the highest median TMB (45.2 mutations/Mb) compared to other tumor types [110]. High TMB is predictive of response to ICI, although prospective validation is lacking [145]. The impressive and durable responses observed with ICI in CSCC are thought to be due to high TMB representing a large number of immunostimulatory neoantigens.

## 6. Tumor Mutational Signatures

Somatic mutations in cancer cells can create a characteristic mutational signature, which reflects the mutational process involved in carcinogenesis. The UV mutation signature—the first characterized signature—is found in the great majority of CSCCs, and even in immunocompromised hosts. UV radiation damage most commonly results in cytosine to thymine or cytosine–cytosine to thymine–thymine changes—i.e., C > T or CC > TT—at dipyrimidine sites [146,147]. Recent studies have also identified signatures in CSCC associated with azathioprine exposure [139] and hyperactivity of endogenous cytidine deaminases (APOBEC)—specifically in patients with epidermolysis bullosa [148].

## 7. Treatment Advances

### 7.1. Localized Resectable High-Risk Disease

Most CSCCs are small, indolent, and surgically resectable, and adjuvant therapy is often not required. Post-operative radiotherapy, however, is considered for patients with resected high-risk localized disease—usually defined as tumors showing involved resection margins, depth of invasion of more than 2–6 mm, extensive perineural invasion, or large nerve involvement [2,149]. Other indications include lymph node involvement and large primary tumors [150,151].

#### 7.1.1. Post-Operative Chemoradiotherapy

Platinum chemotherapy agents such as cisplatin and carboplatin are often used concurrently with postoperative radiotherapy in patients with mucosal head and neck squamous cell carcinoma (HNSCC), as several studies have shown a survival benefit [152,153,154,155]. The results of these trials have been extrapolated and applied to patients with cutaneous SCC. Until recently, there was no definitive prospective study supporting its use in this population.

A phase III trial randomized patients with high-risk resected CSCC to postoperative radiotherapy alone, or with concurrent weekly carboplatin chemotherapy [156]. Concurrent cisplatin is considered the gold standard in HNSCC, but its significant toxicity profile often precludes its use in patients with CSCC who are generally older, and with significant comorbidities. Thus, carboplatin is more frequently used. High-risk disease was defined as patients with primary tumors > 5 cm or T4 disease, resected intra-parotid nodal disease, two or more cervical nodal diseases, or with a node ≥ 3 cm or extranodal extension. Contrary to the results of mucosal HNSCC, no benefit was observed in freedom from locoregional relapse, nor in disease-free or overall survival, in patients with CSCC receiving concurrent chemotherapy. Based on the results of this trial, and the lack of evidence with other regimens, concurrent chemotherapy is generally not recommended in the adjuvant treatment of CSCC outside of clinical trials [151].

#### 7.1.2. Neo/Adjuvant Immunotherapy

The success of ICI in patients with advanced disease has driven efforts to incorporate treatment into earlier stages of disease in order to reduce (a) the morbidity associated with resections of large tumors, and (b) the risk of locoregional relapse or metastasis.

Neoadjuvant immunotherapy is particularly appealing for clinical and translational purposes. Immune activation may be potentiated by the presence of neoantigens and intra-tumoral immune cells within the unresected cancer, and changes in the tumor and stroma can be compared between pretreatment biopsies and the resection specimen [157,158,159]. Furthermore, neoadjuvant studies allow for earlier assessment, using pathologic response, compared to adjuvant studies, where survival data can take many years to mature. A pilot phase II study of two doses of neoadjuvant cemiplimab for patients with locally advanced, curable CSCC resulted in 14/20 patients (70%; 95% CI 45.7–88.1) with a pathological complete response (*n* = 11) or major pathological response (*n* = 3) [160]; this was despite only 30% (95% CI 11.9–54.3) showing a partial response by RECIST, highlighting the challenges of assessing ICI response using current radiological criteria. Neoadjuvant studies of other ICIs, as well as combination neoadjuvant treatment with dual anti-PD(L)1 with anti-cytotoxic T-lymphocyte-associated protein 4 (CTLA-4) blockade, are ongoing [NCT04154943] [161,162]. Furthermore, there are two large phase III adjuvant studies of pembrolizumab or cemiplimab [163,164].

### 7.2. Unresectable Locally Advanced or Metastatic Disease

Historically, no standard of care for systemic therapies existed for patients with unresectable or metastatic CSCC. Cytotoxic chemotherapies such as platinums, fluoropyrimidines, and taxanes have shown activity in retrospective analyses. Response rates are generally low, and the toxicity profiles of therapies often preclude their use in elderly patients with CSCC [165]. ICI with monoclonal antibodies against PD1 and PD-L1 has transformed the treatment landscape for many solid tumors, including CSCC. Other treatment approaches include targeting the epidermal growth factor receptor (EGFR) pathway.

#### 7.2.1. Immunotherapy

A practice-changing phase II study demonstrated the efficacy of cemiplimab—an anti-PD-1 monoclonal antibody—in patients with unresectable or metastatic CSCC [166,167]. Responses were observed in 54.4% (95% CI 47.1–61.6) of patients (both previously treated and untreated) [167]. In patients with initial response, 76% (95% CI 64.1–84.4%) had ongoing response at 24 months, demonstrating the excellent durability of disease response; estimated overall survival at 24 months was 73.3% (95% CI 66.1–79.2) [167,168,169]. As a result of this study, cemiplimab was approved by the FDA, and became the standard treatment for patients with locally advanced or metastatic CSCC who are not candidates for curative surgery or radiation.

Other ICIs, such as pembrolizumab, have shown comparable activity. Two studies—CARSKIN, and KEYNOTE-629—assessed the efficacy of pembrolizumab in advanced CSCC. The objective response rate was 34.3% (95% CI 25.3–44.2%) in KEYNOTE-629 in a heavily pretreated population, and median overall survival has not been ascertained [170]. Based on KEYNOTE-629, pembrolizumab has also been approved by the FDA for advanced CSCC.

The CARKSIN study enrolled treatment-naïve patients with unresectable or metastatic CSCC to receive pembrolizumab [171]. Response rate (RR) at 15 weeks was the primary objective of the study, and was 41% (95% CI 26–58%), including 13 partial and 3 complete responses. Similarly, nivolumab has shown robust results in a phase II first-line study of patients with advanced CSCC [172]. Recently, real-world data regarding the use of ICIs in 245 patients—including immunocompromised patients—were reported to be comparable to trial data [22]. The estimated 12-month OS was 63% (95% CI 51–70); 50% of patients achieved a complete response or partial response (95% CI 44–57), and there were no unexpected toxicities. In univariate and multivariate analysis, ECOG score > 2 was the only clinical factor that was significantly associated with poor OS and PS in the first 6 months.

More aggressive approaches are also being considered for select patients with unresectable localized disease where cure may be possible. A phase II study of neoadjuvant avelumab, followed by curative-dose radiotherapy with concurrent avelumab, is ongoing [173].

Combination strategies of ICIs with cetuximab and oncolytic viruses are being investigated in order to address the challenges of resistance and improve durability of response. CSCC and melanoma share similar features, such as chronic UV exposure and high TMB. Studies of immunotherapy have been established longer in melanoma than in CSCC; thus, approaches that are efficacious or promising in melanoma are being tested in patients with CSCC. Talimogene laherparepvec (T-VEC) is a modified attenuated oncolytic herpes simplex virus containing the granulocyte macrophage colony-stimulating factor (GM-CSF) gene. Production of intratumoral GM-CSF can induce cellular immunity, and the direct oncolytic effect from viral infection of tumor cells can cause an antitumor response. Early-phase studies in metastatic melanoma show that intralesional injections of T-VEC combined with immune checkpoint blockade resulted in an objective response rate of 39% and 50% with concurrent ipilimumab and pembrolizumab, respectively [174,175,176]. Currently, there are studies in CSCC combining oncolytic viruses such as T-VEC and RP1 with ICI or EGFR antibodies [177,178]. EGFR antibodies, which are discussed in more detail below, are also being investigated in combination with anti-PD(L)1 antibodies. A phase II trial will randomize immunocompetent patients with unresectable/metastatic CSCC to avelumab alone, or in combination with cetuximab [179].

#### 7.2.2. EGFR Pathway Inhibition

EGFR is a transmembrane glycoprotein with an extracellular binding domain, along with an intracellular tyrosine kinase domain that regulates cell proliferation via pathways such as MAPK and PI3K. The EGFR protein is highly expressed in CSCC [180,181,182]. EGFR monoclonal antibodies such as cetuximab and panitumumab have shown activity in CSCC in small phase II trials. In a phase II trial of cetuximab in patients with locally advanced unresectable or metastatic CSCC, 28% achieved a response, while 41% had stable disease [183]. An ORR of 31% was observed in a study of panitumumab [184].

Cetuximab has also been used in the neoadjuvant setting. Five out of nine patients receiving neoadjuvant cetuximab alone had a response that allowed for surgical resection and, of these, three had a complete pathological response [185].

Oral tyrosine inhibitors such as gefitinib and dacomitinib, which target the intracellular tyrosine kinase domain, are typically used in patients with EGFR-driven non-small-cell lung cancer, where responses are seen in approximately 75% of patients. These agents have activity in CSCC, with overall response rates of 16% and 28% observed in early phase trials of gefitinib and dacomitinib, respectively [186,187,188].

Combination therapies with drugs known to target common EGFR resistance mechanisms such as fibroblast growth factor receptor (FGFR) signaling are also being investigated. A phase I study of cetuximab with lenvatinib—a multitarget tyrosine kinase inhibitor that has activity against FGFR—in patients with metastatic CSCC or HNSCC is underway [189].

#### 7.2.3. Other Approaches

The risk of BRAF-induced CSCC is abrogated with the addition of MEK inhibition, forming the rationale for investigating the potential role of MEK inhibition in the treatment of CSCC. In vivo studies have shown that MEK induces CSCC cell senescence, but not apoptosis. Interestingly, MEK inhibition also significantly delayed or prevented CSCC development in murine models [190]. Currently, there is a phase II study investigating the efficacy of cobimetinib—an MEK inhibitor—with atezolizumab [191].

Future therapeutic approaches may include novel small molecule inhibitors of both PI3K and mTOR. The oral dual PI3K/mTOR inhibitors—GDC-0084 and LY3023414—have been shown to inhibit proliferation and promote apoptosis in CSCC cell lines [192,193]. GDC-0084 and LY3023414 have been shown to be safe and tolerable in early-phase studies in patients with solid tumors, and there were promising signals of activity [194,195].

## 8. Therapeutic Options for Immunocompromised Patients

Immunocompromised patients have historically been excluded from clinical trials, but with the success of ICI for immunocompetent patients with CSCC, it became apparent that high-level data to guide treatment for immunocompromised patients were lacking. There are several ongoing studies investigating approaches in different groups of immunocompromised patients. A major concern with the use of ICIs in solid organ transplant patents is graft rejection. Case reports and case series have reported up to a 40% risk of graft rejection with the use of anti-PD(L)1 antibodies [196,197]. To potentially ameliorate that risk, two studies are investigating the combination of tacrolimus—an immunosuppressant—with ipilimumab plus nivolumab, and sirolimus with cemiplimab [198]. Tacrolimus and sirolimus—both mTOR inhibitors—may reduce the risk of CSCC development, as discussed earlier in the review. Cemiplimab is also being investigated in patients with CLL, HIV, or allogenic hematopoietic stem cell transplants [199,200].

Recently, real-world data of patients with CSCC receiving cemiplimab included a cohort of immunocompromised patients. Somewhat surprisingly, given the poor prognosis of immunocompromised patients with CSCC compared to immunocompetent patients, ORR and OS did not differ between immunocompetent and immunocompromised patients. Several patients experienced graft rejection as expected. The causes of immunosuppression in this cohort were heterogeneous, ranging from CLL to OTRs and patients with HIV.

Given the increased risk of developing CSCC and its increased lethality in immunocompromised patients, there is an urgent need to better understand the underlying biology driving this disparity, and to identify potential novel treatment approaches for this cohort.

## 9. Conclusions

Our understanding of the underlying biology of CSCC—such as the mechanisms and sequelae of UV-induced DNA damage—has resulted in significant advances in the management of patients with CSCC. ICI is established as the first-line management of advanced CSCC, but focus has now shifted to more challenging questions. Can we reduce the risk of patients with localized disease developing recurrent or metastatic disease? How can we improve current treatment paradigms, particularly in immunocompromised patients, where the risk of treatment-related adverse events—particularly graft rejection in OTRs—is high? Finally, what do we do for patients who do not respond to—or have progressed despite—immunotherapy? Current and future scientific research efforts towards identifying predictive biomarkers and understanding the biology behind clinically disparate groups will hopefully address these clinical challenges.
